# Frequency of respiratory virus-associated infection among children and adolescents from a tertiary-care hospital in Mexico City

**DOI:** 10.1038/s41598-023-47035-6

**Published:** 2023-11-13

**Authors:** Brenda Nieto-Rivera, Zeus Saldaña-Ahuactzi, Israel Parra-Ortega, Alejandro Flores-Alanis, Ebzadrel Carbajal-Franco, Armando Cruz-Rangel, Stephania Galaviz-Hernández, Benjamín Romero-Navarro, Daniela de la Rosa-Zamboni, Marcela Salazar-García, Carmen A. Contreras, Fernando Ortega-Riosvelasco, Irma López-Martínez, Gisela Barrera-Badillo, Hector Diaz-Garcia, Mariana Romo-Castillo, Sarbelio Moreno-Espinosa, Victor M. Luna-Pineda

**Affiliations:** 1 Departamento de Laboratorio Clínico, Hospital Infantil de México Federico Gómez, Ciudad de México, México; 2 Centro de Investigación en Biotecnología Aplicada, Instituto Politécnico Nacional, Santa Inés Tecuexcomac, Tepetitla de Lardizábal, Tlaxcala México; 3 Departamento de Microbiología y Parasitología, Facultad de Medicina, Universidad Nacional Autónoma de México, Ciudad de México, México; 4 Laboratorio de Bioquímica de Enfermedades Crónicas, Instituto Nacional de Medicina Genómica, Ciudad de México, México; 5 Subdirección de Servicios Auxiliares de Diagnóstico, Hospital Infantil de México Federico Gómez, Ciudad de México, México; 6 Subdirección de Atención Integral al Paciente, Hospital Infantil de México Federico Gómez, Ciudad de México, México; 7 Laboratorio de Biología del Desarrollo y Teratogénesis Experimental, Hospital Infantil de México Federico Gómez, Ciudad de México, México; 8 Facultad de Medicina, Universidad Privada Antenor Orrego, Trujillo, Peru; 9 Departamento de Epidemiología Clínica, Hospital Infantil de México Federico Gómez, Ciudad de México, México; 10 Dirección de Diagnóstico y Referencia, Instituto de Diagnóstico y Referencia Epidemiológicos, Ciudad de México, México; 11 Laboratorio de Virus Respiratorios, Instituto de Diagnóstico y Referencia Epidemiológicos, Ciudad de México, México; 12 Centro de Investigación en Malformaciones Congénitas, Hospital Infantil de México Federico Gómez, Ciudad de México, México; 13 Escuela Superior de Enfermería y Obstetricia, Instituto Politécnico Nacional, Ciudad de México, México; 14 Laboratorio de Investigación en COVID-19, Laboratorio de Investigación en Inmunología y Proteomica, Hospital Infantil de México Federico Gómez, Ciudad de México, México; 15 Dirección de Enseñanza, Hospital Infantil de México Federico Gómez, Ciudad de México, México

**Keywords:** Biochemistry, Biological techniques, Immunology, Microbiology, Molecular biology, Medical research, Molecular medicine, Signs and symptoms

## Abstract

Acute respiratory infections (ARIs) are a major cause of morbidity and mortality among children. The causative pathogens show geographic and seasonal variations. We retrospectively evaluated the frequency and seasonality of respiratory pathogens in children and adolescents (age: 0–19 years) with ARIs treated between January 1, 2021, and March 31, 2022, at a single center in Mexico. Out of 2400 patients, 1,603 were diagnosed with SARS-CoV-2 infection and 797 were diagnosed with other common respiratory pathogens (CRPs). Of the 797 patients, 632 were infected with one CRP and 165 with > 2 CRPs. Deaths occurred only in SARS-CoV-2-infected patients. Rhinovirus/Enterovirus, respiratory syncytial virus B, and parainfluenza virus 3 were the most prevalent in cases with single and multiple infections. CRP showed a high frequency between autumn and winter of 2021, with higher incidence of hospitalization compared to COVID-19. The main comorbidities were immunosuppression, cardiovascular disease (CD), and asthma. The frequency of CRPs showed a downward trend throughout the first half of 2021. CRPs increased in single- and co-infection cases between the fourth and fifth waves of COVID-19, probably due to decreased nonpharmaceutical interventions and changes in diagnostic tests. Age, cyanosis (symptom), and immunosuppression (comorbidity) were found to differentiate between SARS-CoV-2 infection and CRP infection.

## Introduction

Acute respiratory infections (ARIs) are the leading cause of mortality in children worldwide, particularly in developing countries^1^. Additionally, ARI is a leading cause of outpatient visits and hospitalization in all age categories^2^. Viruses are the most common pathogens causing ARIs. Respiratory syncytial virus (RSV), rhinovirus/enterovirus (Rhino/Entero), human metapneumovirus (HMPV), parainfluenza virus (PIV), influenza virus (Flu), human coronavirus (CoV), adenovirus (AdVs), and human bocavirus (HboV) account for approximately 70% of all viral ARIs^3^. Atypical pathogens, such as *Mycoplasma pneumoniae* (*M. pneumoniae*), *Chlamydophila pneumoniae* (*C. pneumoniae*), and *Legionella pneumophila* (*L. pneumophila*) are a major cause of bacterial-associated pediatric respiratory infections^4–6^. In addition, patients with viral respiratory infections commonly have bacterial co-infection. For example, studies have documented co-infection of severe acute respiratory syndrome coronavirus 2 (SARS-CoV-2) with *C. pneumoniae* or *M. pneumoniae*^7^.

Since the start of the ongoing coronavirus disease 2019 (COVID-19) global pandemic, a series of nonpharmaceutical interventions (NPIs), such as mandatory use of face masks, promotion of hand hygiene, and social distancing in public spaces, have been implemented to prevent the transmission of SARS-CoV-2^8,9^. These pandemic measures likely helped avoid infection with other respiratory pathogens. For example, in a study conducted in Northern Spain, usual seasonal respiratory viruses, such as RSV, Rhino/Entero, HMPV, PIV, Flu, CoV, and AdVs, were found nearly absent in the pediatric population between 2018–2019 and 2020–2021^10^. In Mexico, national indicators showed a decreased incidence of Flu infection after the implementation of NPIs on March 23, 2020^11^. A low incidence of SARS-CoV-2, Flu, and RSV was reported in Germany during the second and third waves of the COVID-19 pandemic, while Rhino/Entero and AdVs showed a high incidence^12^. Besides, a low incidence of infection with Rhino/Entero, RSV, and AdVs was reported during the second, third, and fourth waves in Colombia^13^. As expected, after the withdrawal of NPIs in Germany, there was a marked resurgence in the incidence of CoVs, HboV, PIV, and RSV infections^14^.

During the ongoing COVID-19 pandemic, respiratory pathogen-associated infections continue to pose a significant health problem. After the relaxation of the NPIs during the COVID-19 pandemic, the pattern of respiratory infections showed changes in pathogen species, temporality, and frequency. However, whether these changes are local or global is an important question that needs to be answered. Thus, this study aimed to evaluate the frequencies of respiratory tract pathogens detected by a respiratory pathogen panel (RPP) and reverse-transcription (RT)-quantitative polymerase chain reaction (qPCR) test in patients aged 0 to 19 years who were treated at a hospital in Mexico City.

## Results

**Figure 1:**
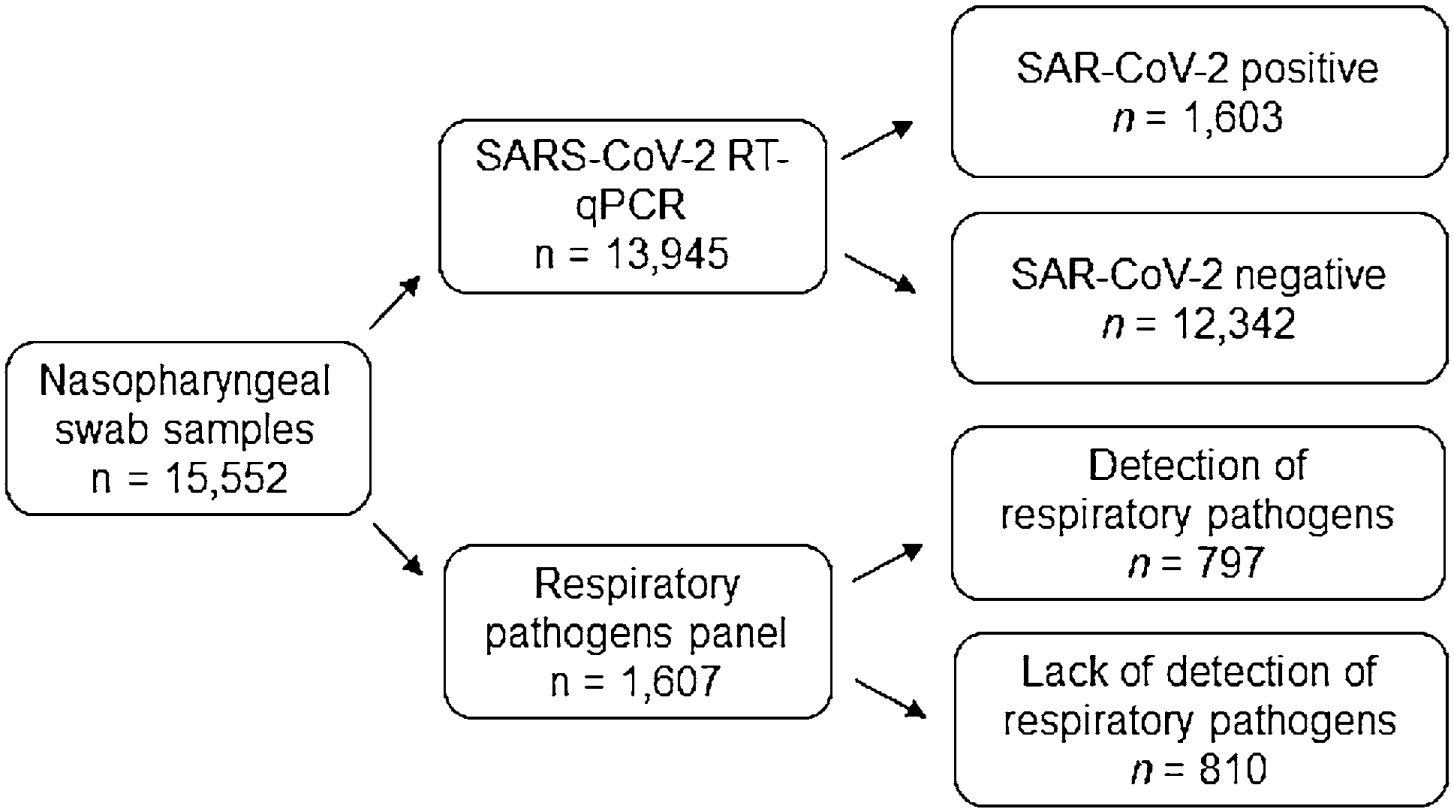
Schematic illustration of the study population. RT-qPCR, reverse-transcription-quantitative polymerase chain reaction. A cycle threshold value of < 40 for SARS-CoV-2 genes and < 35 for internal control was considered indicative of positive test for SARS-CoV-2. The respiratory pathogens were evaluated with NxTAG^®^ Respiratory Pathogen Panel, where the Integrated multiplex PCR and bead hybridization was performed using end-point PCR and read on the NxTAG-Enabled MAGPIX^®^ System.

Of the 15,552 nasopharyngeal swab samples tested at the HIMFG between January 2021 and March 2022, 13,945 (89.67%) samples were tested for SARS-CoV-2 and 1,607 (10.33%) were tested for respiratory pathogens by the RPP (Fig. 1). A total of 1,603 children and adolescents with SARS-CoV-2 and 797 cases of respiratory infection by other viruses were diagnosed in HIMFG (Fig. 1). Of note, the highest detection rate (49.59%) was observed in patients tested for any other respiratory viruses (Table 1).

**Table 1:** Respiratory pathogens detected in the study population.

Respiratory pathogens	*N*	Detection rate (%)
SARS-CoV-2 (RT-qPCR)	1603	11.50
Rhino/Entero	413	11.47
RSV B	182	5.05
PIV 3	107	2.97
HMPV	73	2.03
AdVs	55	1.53
IH3	42	1.17
HboV	42	1.17
Flu A	39	1.08
CoV 229E	20	0.56
RSV A	19	0.53
PIV 4	8	0.22
CoV NL63	5	0.14
CoV OC43	3	0.08
Flu B	1	0.03
IH1	1	0.03
PIV 2	0	0.00
PIV 1	0	0.00
CoV HKU1	0	0.00
*C. pneumoniae*	0	0.00
*M. pneumonia*	0	0.00
*L. pneumophila*	0	0.00

*RT-qPCR* reverse-transcription-quantitative polymerase chain reaction, *RPP* respiratory pathogen panel, *RSV* respiratory syncytial virus, *Rhino/Entero* rhinovirus/enterovirus, *HMPV* human metapneumovirus, *PIV* parainfluenza virus, *Flu* influenza virus, *IH3* influenza A virus subtype H3, *IH1* influenza A virus subtype H1, *CoV* human coronavirus; *AdVs*, adenovirus, *HboV* human bocavirus.

Among the cases tested by the RPP, Rhino/Entero (11.47%), RSV (5.05%), and PIV 3 (2.97%) showed the highest incidence. None of the cases tested positive for PIV 2, PIV 1, CoV HKU1, or bacteria (*C. pneumoniae*, *M. pneumoniae,* or *L. pneumophila*) (Table 1).

### Seasonality of respiratory pathogens

**Figure 2:**
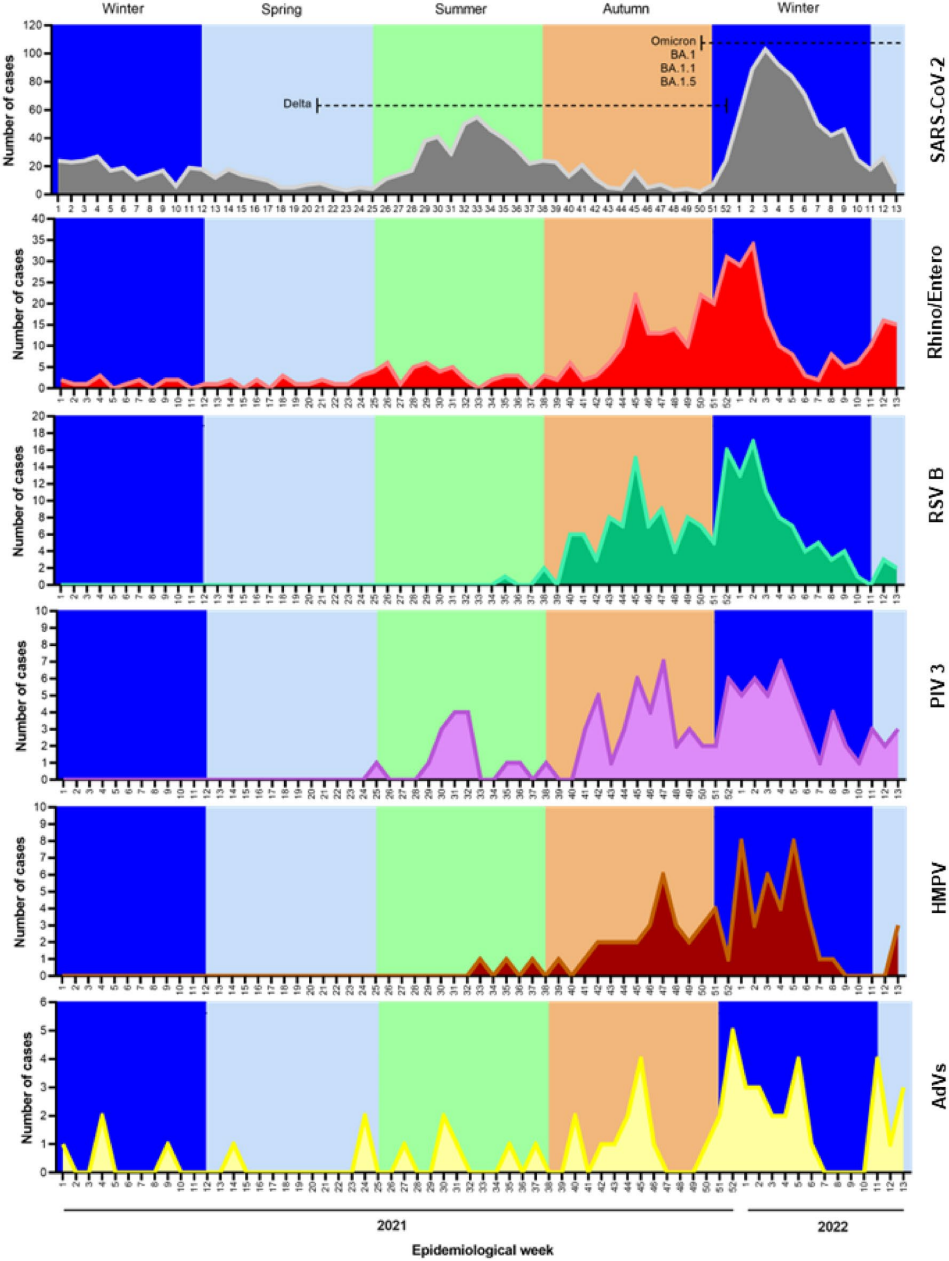
Temporal frequencies of respiratory pathogens detected at the Hospital Infantil de México Federico Gomez (HIMFG). The frequency distribution is shown according to epidemiological week. Different colors indicate different seasons: blue indicates winter, cyan indicates spring, green indicates summer, and brown indicates autumn.

We showed the frequency of respiratory pathogens through SARS-CoV-2 RT-qPCR and RPP in children and adolescents at the HIMFG. The study period included five seasons: winter (from January 1 to March 20, 2021); spring (from March 21 to June 20, 2021); summer (from June 21 to September 25, 2021); autumn (from September 26 to December 26, 2021); winter from December 27, 2021, to March 20, 2022 (Fig. 2).

Although SARS-CoV-2 became more prevalent over time, the detection of SARS-CoV-2 and other CRPs was the lowest during winter-spring 2021, which coincided with strict implementation of infection control and social distancing measures. There were two peaks of SARS-CoV-2 incidence: the first was in summer 2021, which corresponded to the presence of the Delta Variant of Concern (VOC), and the second was in winter 2022, which coincided with the emergence of the Omicron VOC (Fig. 2).

Interestingly, between the SARS-CoV-2 peaks (autumn 2021 and early winter 2021), there was an increase in the detection of other respiratory viruses. With the decrease in the incidence of Omicron VOC, there was a recurrence in the incidence of non-SARS-CoV-2 viruses, principally Rhino/Enterovirus (Fig. 2). Other viruses such as RSV B, PIV 3, HMPV, AdVs, IH3, HboV, CoV 229E, and RSV A showed an increasing trend from the beginning of the summer of 2021 and the winter of 2022, the periods corresponding to the fourth and fifth waves of COVID-19, respectively (Fig. 2 and Supplementary Fig. 1).

Additionally, the highest detection rate was registered in the autumn of 2021 (51.27%), which was significantly higher than that in the winter (p = 0.0001) and spring (p = 0.0035) of the same year and the winter of 2021–2022 (p = 0.0249), but not significantly higher than that in the summer of 2021 (p = 0.6674) and the spring of 2022 (p = 0.8466) (Table 2).

**Table 2:** Seasonality of respiratory pathogens detected in the study population.

Year	Date	Season	Test #	Infection (DR%)	Co-infection (DR%)	Multiple-infection (DR%)
2021	Jan 1–Mar 20	Winter	72	14 (19.44)	3 (4.17)	0 (0)
	Mar 21–Jun 20	Spring	70	18 (25.71)	4 (5.71)	0 (0)
	Jun 21–Sep 25	Summer	117	55 (47.01)	11 (9.40)	1 (0.85)
	Sep 26–Dec 19	Autumn	394	202 (51.27)	48 (12.18)	2 (0.51)
2022	Dec 20–Mar 20	Winter	879	306 (34.81)	80 (9.10)	7 (0.80)
	Mar 21–31	Spring	75	37 (49.33)	9 (12.00)	0 (0)
		Total	1607	632 (39.33)	155 (9.65)	10 (0.62)

*DR%* detection rate percentage.

### Co-infections of different respiratory pathogens

**Figure 3:**
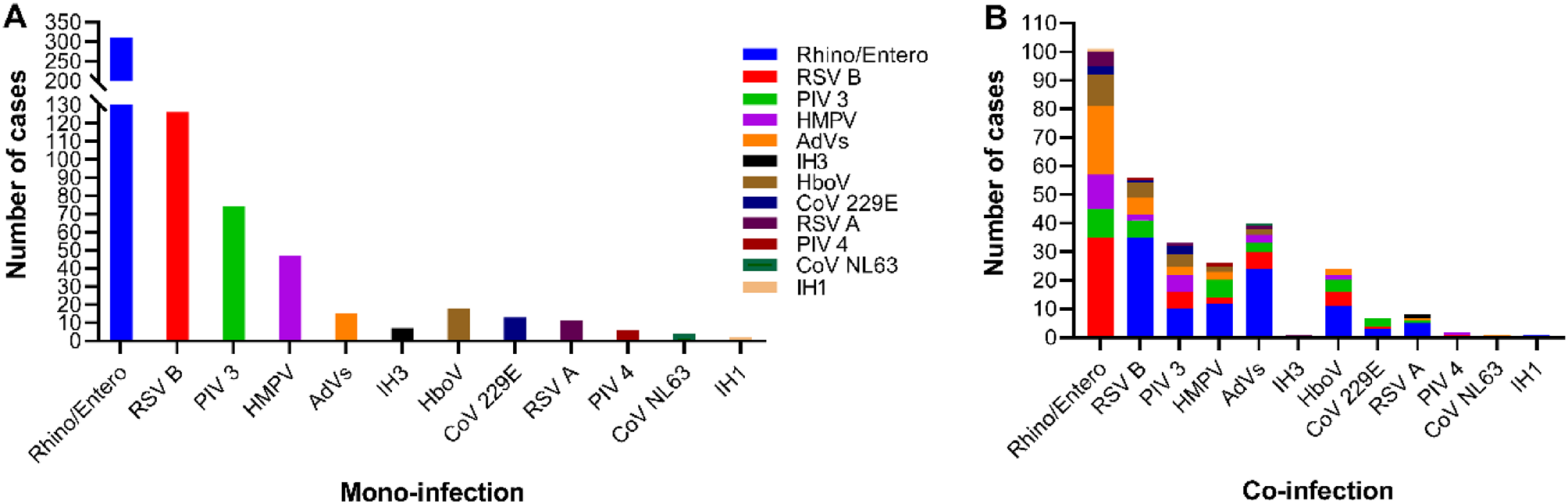
Cases of mono- and co-infections detected by respiratory pathogens panel. (**A**) Number of cases of mono-infection; (**B**) number of cases of co-infection (two viruses); the x axis is the respiratory virus, and each color indicates the co-infecting virus. *RSV* respiratory syncytial virus, *Rhino/Entero* rhinovirus/enterovirus, *HMPV* human metapneumovirus, *PIV* parainfluenza virus, *IH3* influenza A virus subtype H3, *IH1* influenza A virus subtype H1, *CoV* human coronavirus, *AdVs* adenovirus, *HboV* human bocavirus.

Among the 797 cases tested by the RPP, 632 (79.29%) were infected with one respiratory pathogen, and 155 (19.44%) were infected with two respiratory pathogens. Rhino/Entero had the highest co-infection rate with RSV B (35/101, 34.6%), AdVs (24/101, 23.8%), HMPV (12/101, 11.9%), HboV (11/101, 10.9), PIV 3 (10/101, 9.9%), RSV A (5/101, 4.9%), CoV 229E (3/101, 2.9%), and IH1 (1/101, 0.9%). Analysis of co-infections with RSV B revealed PIV 3 and AdVs as having the highest frequency (28.57% each) (Fig. 3).

Among cases with co-infections, a significant association was observed between Rhino/Entero and RSV B (OR 1.94, 95% confidence interval (CI) 0.99–3.87; *p* < 0.0417). None of the other viruses showed any significant association with each other.

No co-infections of SARS-CoV-2, PIV 2, PIV 1, CoV, and HKU1 were found in our cohort.

**Figure 4:**
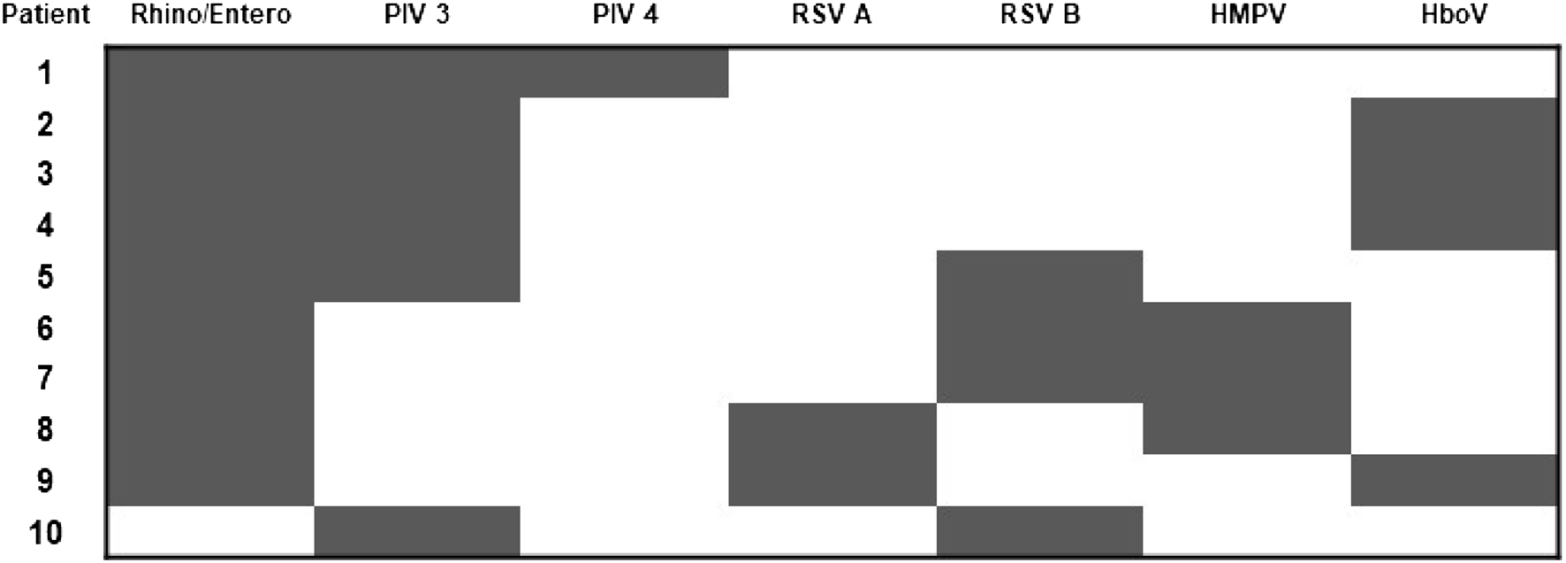
Cases of multiple infections (three viruses) detected by RPP. *RSV* respiratory syncytial virus, *Rhino/Entero* rhinovirus/enterovirus, *HMPV* human metapneumovirus, *PIV* parainfluenza virus, *HboV* human bocavirus.

Fifteen patients (1.88%) in this study showed simultaneous infection with three respiratory pathogens; among these, Rhino/Entero-PIV 3-HboV were the most common concomitant infections (3/15, 20%), followed by Rhino/Entero-RSV B-HMPV (2/15, 13.3%) (Fig. 4).

### Demographic and clinical features

All patients (N = 15,552) were attended and tested, nevertheless, only positive patients for SARS-CoV-2 (n = 1,603) and other viruses (n = 797) were ambulatory treated or hospitalized. The mean age of patients with COVID-19 was 8.14 (± 5.62) years; the mean age of patients with mono-infection, co-infection, and multi-infection for CRPs was 5.82 (± 5.06), 4.97 (± 4.32), and 2.45 (± 2.02) years, respectively (Table 3). The study population was predominantly male, and the majority were treated as outpatients. Patients with COVID-19 had a significantly higher incidence of intubation (p < 0.0001) (Table 3). Eleven children with COVID-19 died during May to December 2020.

**Table 3:** Demographic and clinical features of the study population.

	COVID-19 ^Ref^	Respiratory virus infection		
	SARS-CoV-2 (n = 1,349)	Mono-infection (n = 628)	Co-infection (n = 155)	Multi-infection^§§§^ (n = 10)
Sex^§^				
Male	53.04	56.37	60.00	60.00
Female	46.96	43.63	40.00	40.00
Age (years)^§§^	8.14 (± 5.62)	5.82 (± 5.06)***	4.97 (± 4.32)****	2.60 (± 2.07)**
Patient care^§§§^				
Ambulatory	62.71	59.39	63.87	70.00
Hospitalized	37.29	40.61	36.13	30.00
Patient status				
Intubated^§§§^	2.59	0.16****	1.20	0.00
ICU^§§§^	3.78	3.18	1.20	0.00
Deaths	0.82	0.00*	0.00	0.00
Comorbidities				
Diabetes mellitus^§§§^	0.67	0.96	1.29	10.00
COPD^§§§^	0.15	0.16	0.65	0.00
Asthma^§^	2.67	9.24****	5.81*^(§§§)^	10.00
Chronic hypertension^§^	1.19	2.87*	2.58	0.00
Obesity^§^	3.11	1.43*	1.94	0.00
Chronic kidney disease^§^	2.67	2.23	1.94	0.00
Immunosuppression^§^	9.34	19.27****	12.9	20.00
HIV^§§§^	0.22	0.16	1.94*	0.00
CD-NH^§^	3.56	6.37**	7.74*	10.00
Passive smoker/smoking^§§§^	0.67	0.48	0.00	0.00
Other diseases^§§§^	4.67	0.64****	0.65*	0.00
Sings and symptoms^§^				
Fever	43.96	47.13	59.35***	60.00
Cough	31.13	55.89****	63.23****	60.00
Odynophagia	10.75	10.99	9.03	0.00
Dyspnea	12.31	20.06****	18.06	30.00
Irritability	17.05	19.43	14.19	40.00
Diarrhea	12.38	10.19	7.10	10.00
Thoracic pain	8.15	6.37	6.45	0.00
Chills	12.23	5.73****	10.32	0.00
Headache	18.09	14.65	11.61	0.00
Myalgia	10.38	3.18****	4.52*	0.00
Arthralgia	7.56	2.07****	3.23****	0.00
Rhinorrhea	15.05	28.18****	30.97	40.00•
Polypnea	8.82	11.62	12.26	10.00
Vomiting	12.82	8.60**	8.39	20.00
Abdominal pain	13.27	9.55*	4.52**	10.00
Conjunctivitis	4.67	5.73	4.52	10.00
Cyanosis	3.19	43.79****	47.74****	50.00****
Anosmia^§§§^	3.56	0.48****	1.94	0.00
Dysgeusia^§§§^	2.89	0.32****	1.29	0.00

*ICU* intensive care unit, *HIV* human immunodeficiency virus, *COPD* chronic obstructive pulmonary disease, *CD-NH* cardiovascular disease (no hypertension).

^§^Pearson's *Chi*-squared test with Yates' continuity correction.

^§§^Kruskal-Walli’s rank sum test with *post-hoc* Bonferroni correction. Normality of distribution was assessed using the Shapiro–Wilk normality test (*p* < 0.05).

^§§§^Exact Fisher for categorical variables. For comparisons the SARS-CoV-2 column was taken as the reference (ref).

Statistically significant differences were considered when the *p*-values were < 0.05 (*), < 0.01 (**), < 0.001 (***), and < 0.0001 (****).

The HIMFG is a tertiary-care pediatric hospital that admits a large population of children and adolescents with underlying diseases. Immunosuppression, asthma cardiovascular disease were the main ARIs-associated comorbidities, while obesity showed an association with COVID-19. The main symptoms of COVID-19 and infections with CRPs were fever and cough. However, cyanosis (p < 0.0001) was the representative sign of infection with CRPs.

## Discussion

ARIs are a set of infectious diseases of the respiratory system including common cold, pneumonia, otitis, tonsillitis, sinusitis, acute bronchitis, laryngotracheitis, bronchiolitis, and laryngitis. ARIs have an evolution period of less than 15 days and are characterized by the presence of one or more clinical symptoms or signs such as cough, rhinorrhea, nasal obstruction, odynophagia, otalgia, dysphonia, noisy breathing, respiratory distress, which may or may not be accompanied by fever^15^. In this study, we reported the detection rate of 15 CRPs among patients aged 0–19 years who had symptoms of ARI and received medical attention at HIMFG. The main causative agents in our cohort were viruses, with Rhino/Entero and RSV B being the most prevalent. These findings are consistent with those of other studies, in which the same viruses were identified as the most common pathogens in children and, in particular, infants^16–18^. The detection rate of viruses found in this study is consistent with results from similar studies conducted in Cabo Verde^16^, Dhaka, Bangladesh^19^, Ghana^20^, and Argentina^21^. However, higher rates of viral detection have been reported from other locations worldwide^17,22^. These differences could be attributed to different climatic conditions, enrollment criteria, case definitions, and testing platforms used for diagnosis. The studies conducted in Naples and Niger pertained to the pre-COVID-19 pandemic^17,22^. There are several multiplex assays available for the detection of respiratory pathogens. Of these, Luminex NxTAG Respiratory Pathogen Panel has shown a high accuracy along with the FilmArray^®^ Respiratory Panel (BioFire) and GenMark eSensor RVP^23^. It is pertinent to mention that in Mexico, the testing platforms were implemented during the COVID-19 pandemic. Before this period, influenza and RSV were the only viruses for which PCR was performed in symptomatic patients. Moreover, the number of diagnostic tests for respiratory viruses performed during the first quarter of 2022 was higher than that in 2021. Of note, the decision to perform viral panel diagnostic tests was at the discretion of the medical staff.

At the end of 2020 and the first half of 2021, Mexico was going through the most critical phase of infections and deaths related to COVID-19 (https://coronavirus.jhu.edu/region/mexico). Therefore, the epidemiological surveillance strategy implemented by the Mexican government included suspected cases (based on the clinical picture or an epidemiological association) and confirmed cases based on any of the following three processes: test confirmed by an authorized laboratory, epidemiological association with a case confirmed by a laboratory test, and cases confirmed by an opinion committee (only applicable for postmortem diagnosis)^24^. This implied that patients showing more than one symptom associated with SARS-CoV-2 infection were diagnosed as a probable case of COVID-19 without requiring a diagnostic test. Since the symptoms of other respiratory viral infections are similar to those of SARS-CoV-2 infection, it is unsurprising that most patients had developed a respiratory virus infection instead of COVID-19. A few cases were evaluated by RT-qPCR for SARS-CoV-2 and NxTAG® Respiratory Pathogen Panel, but none of these cases showed co-infection of SARS-CoV-2 and other CRPs.

The NPIs implemented during the COVID-19 pandemic altered the community circulation of other human respiratory pathogens (viruses and bacteria). These measures helped decrease infectivity leading to a reduction in the number of hospitalizations and deaths caused by non-COVID-19-associated respiratory infections^9^. Of note, social distancing and the use of face masks during the pandemic also reduced the transmissibility of these pathogenic microorganisms^25^.

In this study, the frequency of respiratory viruses showed a downward trend throughout the first half of 2021. This was likely attributable to the implementation of social distancing and mandatory use of face masks by the national government during the pandemic, which helped reduce the exposure of the pediatric population to the classic respiratory viruses that affect this population segment.

The seasonality of respiratory pathogens is still a matter of study^26^. Humidity, temperature, and pollution are known to modulate the host response, and human behavior and physiological factors may also be involved^27^. Classically, cold weather and low relative humidity are associated with the onset of some viral ARIs^28,29^. Mexico has temperate weather, and it has been reported that RSV peaks in autumn–winter, AdVs in spring, while Flu, PIV, and HMPV peak in winter^26^. Nevertheless, we observed two peaks of CRPs in our study, i.e., in autumn (2021) and winter (2022). A study conducted in Mexico found that the average number of seasonal influenza cases during the COVID-19 pandemic decreased by 64% compared to the number in the pre-pandemic years, which was likely attributable to the containment measures implemented to control SARS-CoV-2 infection^11^. This phenomenon was also observed in other cities worldwide^30–33^. It has long been recognized that Flu, RSV, and CoV outbreaks occur in temperate climates during winter, whereas low activity is detected during summer^34^. However, in the second half of 2021, before the onset of winter, with the implementation of a massive vaccination campaign against SARS-CoV2 and the relaxation of prevention measures, an increase in the frequency of cases of respiratory viral infection was observed^35^. Fourteen types of respiratory viruses have been shown to affect the Mexican pediatric population, which had maximum activity during the winter season (October–February)^36,37^. In the present study, we observed a resurgence in the incidence of respiratory viral infections in mid-October 2021, reaching the peak during the first half of January 2022. This suggests that these viruses did not present atypical activity, despite the peaking of infections of the omicron variant of SARS-CoV-2 during this period. A previous study also showed no significant change in the incidence of respiratory viral infections during the influenza pandemic in 2009 compared to other seasons^38^.

In previous studies conducted in Mexico, Entero/Rhino, RSV, PIV, AdVs, HboV, HMPV, and Flu A and B were found to have the highest incidence in the pediatric population^26,36,39^. In the present study, Entero/Rhino, PIV, HMPV, HboV, and AdVs were the most frequently detected viruses during the winter of 2021–2022, indicating a slight variation in the viral diversity in the presence of SARS-CoV-2.

Co-infections are substantially more common in children, especially under the age of 5 years^40^. Furthermore, RSV was found to increase the risk of lower respiratory tract infection when co-infected with human rhinovirus, HMPV, and PIV 3 but not with influenza A^41^. Further studies are required to explore the clinical relevance of mixed viral infection in the respiratory tract.

Pre-pandemic studies reported lower association of co-infections between respiratory viruses and bacteria, such as Rhino, PIV, Flu A and Flu B^42–44^. Interestingly, no bacterial co-infections were detected in this study. In previous studies, co-infection with *C. pneumoniae* or *L. pneumophila,* two of the three bacteria detected in the RPP, was not detected in patients with COVID-19; however, *M. pneumonia*, another bacteria detected in the RPP, was found to be the most common bacterial co-infection in both pediatric and adult patients with COVID-19^45–48^. Other studies have found a relatively infrequent prevalence of bacterial co-infection in patients with COVID-19^49^. A high rate of bacterial co-infection in COVID-19 patients was reported among patients admitted to the ICU^48,50^. A study comparing the bacterial co-infection with Flu, RSV, and SARS-CoV-2 only showed co-infection between *Mycoplasma spp*. with Flu and *Legionella spp*. with SARS-CoV-2^51^. In a pediatric acute otitis media patient study, no co-infections were identified between *C. pneumoniae*, *L. pneumophila,* RSV, AdV, HMPV, HboV, PIV, and Flu^52^. An essential factor to consider is that many of these bacterial co-infections were reported in pneumonic and hospitalized patients when clinical manifestations are critical, and many times required medical dispositive that make patients vulnerable to opportunist infections. The samples analyzed in this study were collected during early infection stages. All findings may explain this study's lack of detection of bacterial co-infections.

Whether NPIs break the previous epidemic pattern and cause changes in pathogen species or seasonality and whether these changes are local or global are key questions that need to be answered. A better understanding of the role played by NPIs in the transmission dynamics of respiratory viruses can guide future prevention recommendations.

Studies have suggested that lower transmission years are typically followed by a stretch of more aggressive transmission due to decreased antiviral herd immunity^53–56^. Clinicians should be aware that respiratory viruses may not exhibit typical seasonal circulation patterns and that a resumption of circulation of certain respiratory viruses is occurring. Therefore, a high index of suspicion and testing for multiple respiratory pathogens is important for the clinical management of ARIs.

The HIMFG is a tertiary-care pediatric hospital that admits a large population of children and adolescents for surgery, metabolic diseases, organ transplants, and chemotherapy. In this study, the observed association between obesity and COVID-19 was consistent with other studies^57,58^. Another study conducted in Mexico found an association between obesity and a worse COVID-19 prognosis^59^. Immunosuppression, asthma, and cardiovascular disease were the main comorbidities in the study population. An international multicenter study involving 19 hospitals across Russia, Turkey, China, and Spain identified the presence of these comorbidities as a risk factor for infection with the influenza virus^60^. A high number of CRP-infected patients presented cyanosis, which is contrary to other studies that showed no significant association between cyanosis and CRP infection^61,62^. Previous studies have shown that the symptoms of COVID-19 may mimic those of cardiovascular disease, such as cyanosis and breathlessness^63^. However, we did not find a correlation between cyanosis and COVID-19 in this study.

Some limitations of this study should be considered. First, this was a retrospective observational study. Second, this study addressed the SARS-CoV-2 pandemic and was conducted on children and adolescents from a third-level hospital, where children admitted were more likely to suffer from underlying diseases and exhibit a more severe disease course. Thus, our findings may not be entirely generalizable to the general pediatric population. However, this study showed the effect of the SARS-CoV-2 pandemic on CRP-associated infections in children and adolescents. Our findings may help inform more effective interventions to prevent and treat these infections.

## Methods

This was a retrospective observational study conducted at the Hospital Infantil de México Federico Gomez (HIMFG), a tertiary-care hospital for children and adolescents in Mexico City. Between January 1, 2021, and March 31, 2022, a total of 15,552 nasopharyngeal swabs were sampled from patients with respiratory symptoms in accordance with the clinical practice guidelines on respiratory infection. These swabs were tested for SARS-CoV-2 as part of the hospital’s protocol using SARS-CoV-2 RT-qPCR and RPP (Fig. 4). We retrospectively investigated the frequency of respiratory pathogen-associated infections in this population.

All tests were performed at the Central Laboratory of the HIMFG. A molecular test (RT-qPCR) to detect viral genes, including *E*, *N,* and *RdRp*, and human RNAase P gene (internal control), was performed using the GeneFinder™ COVID-19 Plus RealAmp Kit (OSANG Healthcare Co. Ltd., Korea) and QuantStudio™ 5 Real-Time PCR System (Applied Biosystems, USA). A cycle threshold value of < 40 for SARS-CoV-2 genes and < 35 for internal control was considered a positive test of SARS-CoV-2.

The RPP was performed using the NxTAG^®^ Respiratory Pathogen Panel (Luminex Corporation, USA), which contained 21 common respiratory pathogens (CRPs), i.e., Rhino/Entero, RSV A, RSV B, PIV 1, PIV 2, PIV 3, PIV 4, HMPV, HboV, AdVs, Flu A, Flu B, influenza A virus subtype H1 (IH1), influenza A virus subtype H3 (IH3), CoV 229E, CoV NL63 CoV OC43, CoV HKU1, *C. pneumoniae*, *M. pneumonia*, and *L. pneumophila*. The Integrated multiplex PCR and bead hybridization were performed using end-point PCR with a Veriti™ 96-Well Thermal Cycler (Applied Biosystems, USA), while the results were read on the NxTAG-Enabled MAGPIX^®^ System (Luminex Corporation).

The demographic and clinical database was downloaded from the Sistema Nacional de Vigilancia Epidemiológica (https://www.sinave.gob.mx/). All datasets were filtered to retain only patients who tested positive for SARS-CoV-2 or CRPs. Demographic data included sex and age, and clinical data included patient status, comorbidities, and signs/symptoms. The comorbidities were defined by Flores-Alanis et al. and the signs/symptoms were defined by Cortés-Sarabia et al.^24,64^.

The temporality of the appearance of SARS-CoV-2 variants in Mexico was extracted from the studies by Flores-Alanis et al. and Zárate et al.^65,66^. The data on the national vaccination program against COVID-19 by the federal government of Mexico were obtained from https://vacunacovid.gob.mx/ [accessed on February 28, 2022] and the study by Flores-Alanis et al.^24^.

The detection rate per season was calculated by dividing the number of positive tests by the total number of tests applied multiplied by 100. The differences in pathogens in co-infections and differences in positivity rates between seasons were tested using the chi-squared test. The association between viruses in co-infections was assessed by calculating the odds ratio (OR) using a 2 × 2 contingency table and Fisher’s exact test. *p* < 0.05 was considered indicative of statistical significance for all tests. All statistical analyses were performed using the stats v4.1.3 and DescTools v.0.99.45 R packages from RStudio v3.2.2. The graphs were generated using GraphPad Prism version 9.0.0 for Windows (GraphPad Software, San Diego, CA, USA; https://www.graphpad.com) and RStudio v3.2.2.

### Ethics approval and consent to participate

The Central Laboratory of the HIMFG provided the data analyzed in this retrospective analysis. The datasets were obtained from the SARS-CoV-2 test (RT-qPCR) and RPP of children and adolescents previously diagnosed and cared for, according to relevant guidelines and regulations from the HIMFG. The study was reviewed and approved by the Research Committee (Dr. Juan Garduño Espinosa), Ethics Committee (Dr. Miguel Ángel Gaxiola García), and Biosecurity Committee (Dr. Marcela Salazar García) of HIMFG, with permit numbers HIM/2020/029 SSA.11664. Informed consent and settlement were obtained from all patients in the study. All data were anonymized prior to analysis.

## Conclusions

The frequency of CRPs, such as Rhino/Entero, RSV B, and PIV 3, showed a downward trend throughout the first half of 2021. Nevertheless, CRPs increased in single- and co-infection cases between the fourth and fifth waves of coronavirus disease 2019, especially in the autumn and winter of 2021–2022, probably due to decreased NPIs such as mandatory use of face masks, promotion of hand hygiene, and social distancing in public spaces. Hospitalized and intubated patients were mainly found to have mono-infection with CRPs. Fever and cough were the main signs and symptoms of COVID-19 and infections with CRPs, but cyanosis was the representative sign of infection with CRPs.

### Supplementary Information

Supplementary Figure 1.

## Data Availability

The HIMFG data supporting this study’s findings are available from the corresponding author (V.L.) upon reasonable request.

## Abbreviations

ARIs: Acute respiratory infections
CRPs: Common respiratory pathogens
RSV: Respiratory syncytial virus
Rhino/Entero: Rhinovirus/enterovirus
HMPV: Human metapneumovirus
PIV: Parainfluenza virus
Flu: Influenza virus
IH3: Influenza A virus subtype H3
IH1: Influenza A virus subtype H1
CoV: Human coronavirus
AdVs: Adenovirus
HboV: Human bocavirus
COVID-19: Coronavirus disease 2019 (COVID-19)
NPIs: Nonpharmaceutical interventions
RPP: Respiratory pathogen panel
RT: Reverse-transcription (RT)
qPCR: Quantitative polymerase chain reaction
HIMFG: Hospital Infantil de México Federico Gomez
OR: Odds ratio
