# Supplementary material for: Frequency of respiratory virus-associated infection among children and adolescents from a tertiary-care hospital in Mexico City
Source: Sci Rep. 2023 Nov 13;13:19763. doi: 10.1038/s41598-023-47035-6 (PMC10643542; doi:10.1038/s41598-023-47035-6)
Supplement: Supplementary file 1 — Supplementary Figure 1. [file 41598_2023_47035_MOESM1_ESM.docx]

**
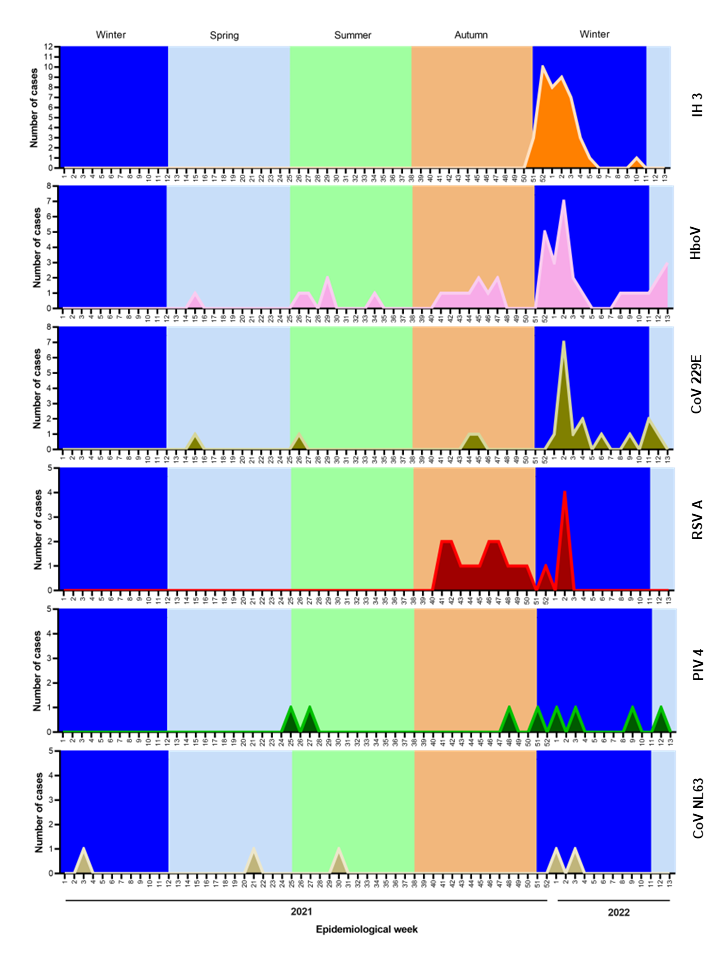
**

**Figure Supplementary 1.** Frequency of the least abundant respiratory pathogens in population attended at the Hospital Infantil de México Federico Gomez (HIMFG). The frequency was distributed as epidemiological week, seasons were showed as blue for winter, cyan for spring, green for summer, and brown for autumn.
